# Funding emergency response

**DOI:** 10.2471/BLT.24.020524

**Published:** 2024-05-01

## Abstract

Protracted humanitarian emergencies are forcing donors and agencies to rethink their approaches to response. Gary Humphreys reports.

Angela Huddleston can see it coming. “We’re going to start feeling the funding crunch from around the second half of 2024,” she says. “I'm genuinely terrified of what it’s going to mean for the communities we work with.”

Deputy country director in the Syrian Arab Republic for the International Rescue Committee (IRC), a humanitarian nongovernmental organization, Huddleston knows exactly how much the communities she serves in the north of the country rely on IRC for support.

That support includes health service provision ranging from primary, secondary and emergency health care to mental health services as well as pharmaceutical supply chain management. “We also work on gender-based violence prevention and economic recovery development, along with programmes devoted to livelihoods access and agriculture support, as well as early childhood development,” Huddleston says.

It is a comprehensive humanitarian package built up over the 13 years that the organization has been in the Syrian Arab Republic. And it is under strain because of the funding crunch Huddleston and her colleagues are now facing.

IRC is not alone. According to the Syrian Arab Republic's Humanitarian Response Plan (HRP) – a strategic framework developed by United Nations (UN) agencies, nongovernmental organizations (NGOs) and other partners designed to help coordination and delivery of humanitarian assistance – less than 40% of the 5.41 billion United States dollars (US$) called for in 2023 was raised, and this year it is likely to be worse.

The worldwide humanitarian funding picture is similarly bleak, according to the UN Global Humanitarian Overview (GHO), an annual exercise that brings together humanitarian organizations, agencies and other partners to assess, prioritize and respond to humanitarian crises around the globe.

In 2023, the GHO resulted in a call for a record US$ 56.1 billion. Only 41.6% of that was raised.

“We are confronting the worst shortfall in humanitarian assistance funding that we have seen,” says Jens Laerke, spokesperson for the Office for the Coordination of Humanitarian Affairs (OCHA), the UN’s coordination agency for humanitarian aid that tracks funding based on inputs from donors and recipient agencies.

According to Laerke, the shortfall reflects trends that have been developing for some time, salient among them the sharp increase in demand. “At the end of 2023, the total amount requested was US$ 56.1 billion, up from US$ 25.1 billion in 2018.”

Laerke identifies multiple drivers of this near-doubling, including the coronavirus disease 2019 (COVID-19) pandemic, climate change-related events such as floods and droughts which have impacted populations around the world, and political instability and conflict.

“We are confronting the worst shortfall in […] funding that we have seen.”

Jens Laerke

In many cases, countries are struggling with all four. “It’s fairly typical to see composite emergencies,” says Sorcha O’Callaghan, director of the humanitarian policy group at the Overseas Development Institute in London, United Kingdom of Great Britain and Northern Ireland. O’Callaghan cites numerous examples, among them Sudan, where a governance crisis and armed conflict are compounding long-term effects of climate change.

Demand is also growing because of what Laerke terms a ‘piling up’ of humanitarian emergencies. “We have a number of emergencies that are now entering their second decade and show no sign of being resolved,” he says. “And instead of providing emergency relief, for example through the provision of temporary shelter, food and water, humanitarians are finding themselves providing basic services such as running health clinics in Yemen, Syria or Afghanistan.”

One likely impact of the current funding crunch is likely to be a paring back to emergency response essentials for many humanitarian actors. 

For example, the International Committee of the Red Cross (ICRC), a humanitarian organization that assists victims of armed conflict and other violence, plans to focus on core activities as a result of cutbacks. In December 2023, the ICRC announced that around 4000 positions were being cut across its global operations in 2023 and 2024, leaving a workforce of approximately 18 500 people. The projected budget for the ICRC in 2024 is 2.1 billion Swiss Francs (US$ 2.4 billion), down from 2.8 billion Swiss Francs (US$ 3.2 billion) in 2023.

According to Daniel Littlejohn-Carrillo, head of resource mobilization for the ICRC, the organization will be focusing on its core missions, namely protecting victims of war and other situations of violence, and talking with warring parties and other actors about their obligations under international humanitarian law.

Whether or not donor funding can pick up again is open to question. “In 2023, the total amount disbursed dropped for the first time, falling from US$ 30 billion in 2022 to just over US$ 23 billion last year,” Laerke says.

For Laerke, the narrowness of the international donor base is also a matter for concern. “As things stand, more than 50% of multilateral global humanitarian funding comes from the United States of America, Germany and the European Union,” he says. “This not only limits the funds that can be mobilized but makes them subject to adverse events that have particular relevance for those countries, such as the ongoing hostilities in Ukraine and Gaza.

“I'm genuinely terrified.”

Angela Huddleston

Huddleston concurs, highlighting the significant shift in humanitarian funding flows away from the Syrian Arab Republic as a result of increasing humanitarian need elsewhere.

“Several of our governmental donors have made a substantial funding cut to their Syria programme in 2024, exacerbating a situation already rendered challenging by the World Food Programme’s decision to cut the number of people reached with food assistance across Syria from 5.5 million in July to 3.0 million by the year end,” she says.

While it is widely accepted that a broader and deeper funding base might reduce such instability, there is a building consensus regarding the need to do more than simply raise more money.

O’Callaghan believes that reform could begin with making the machinery of humanitarian response more efficient. “At the moment we’re having to manage a very complex and bloated system,” she says. “We need to simplify it and make sure that money is spent on operations. There’s also a need to work harder on humanitarian assessments, to support more effective targeting of aid.”

O’Callaghan also believes that more could be done to leverage the power of local resources. “There is tremendous capacity at the local and very local level, and we're still not working effectively with local, national, regional actors,” she says.

Laerke stresses the need for humanitarians to engage with development agencies, acknowledging that many crises could be mitigated with more resilient infrastructure and institutions.

Adelheid Marschang, senior emergency officer at the World Health Organization (WHO), like O’Callaghan, favours a cross-sectoral, cross-government and cross-agency approach that has come to be referred in humanitarian circles as the humanitarian, development, peace-building nexus.

“The nexus approach recognizes the composite nature of humanitarian crises and the ways in which the different components interconnect,” Marschang explains. “Humanitarian crises often occur in contexts of protracted conflict or fragility where development has stalled, while development gains are difficult to achieve in the absence of peace and stability.”

ICRC’s Littlejohn-Carrillo also stresses the link between crises and conflict. “The international community needs to take serious steps towards reducing the causes of humanitarian need, starting with reducing the number of armed conflicts,” he says.

For Marschang, advancing the nexus agenda will require engaging with governments at the highest level. She believes donors themselves have a role to play here.

“In Afghanistan we have seen that donors with political influence can make a huge difference,” she says. “So we need targeted, high-intensity advocacy to those partners who can influence funding and political situations in priority countries. In the end, addressing many of these crises comes down to finding political solutions.”

Huddleston could not agree more. “Until we see a permanent political solution to the situation in the Syrian Arab Republic, and in other situations like it, the only option we have is this band-aid approach to humanitarian aid. We owe it to the communities we serve to do better than that.”

**Figure:**
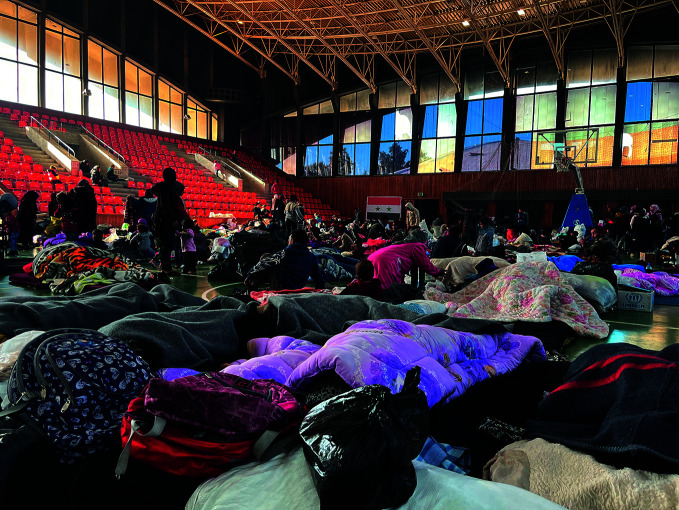
People shelter in Aleppo, Syrian Arab Republic after the February 2023 earthquakes

WHO / Inas Hamam

**Figure:**
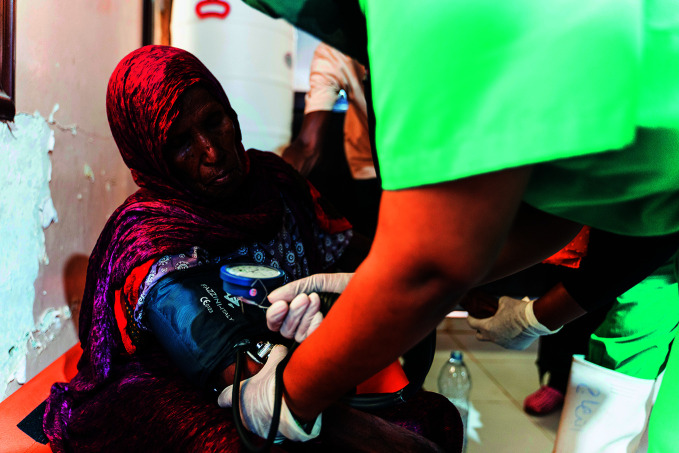
A woman receives care at the cholera treatment centre in Gadarif, Sudan on 23 October 2023

WHO / Ala Kheir

